# Screening and Identification of an Immune-Associated lncRNA Prognostic Signature in Ovarian Carcinoma: Evidence from Bioinformatic Analysis

**DOI:** 10.1155/2021/6680036

**Published:** 2021-04-30

**Authors:** Yan Li, Fan-fan Huo, Ying-ying Wen, Miao Jiang

**Affiliations:** ^1^Clinical Skill Training Center, The Second Hospital of Shandong University, Jinan, Shandong 250033, China; ^2^Department of the Second Operation, The Second Hospital of Shandong University, Jinan, Shandong 250033, China; ^3^Department of the first Operation, The Second Hospital of Shandong University, Jinan, Shandong 250033, China

## Abstract

**Backgrounds:**

The dysregulated long noncoding RNAs (lncRNAs) have been described to be crucial regulators in the progression of ovarian carcinoma. The infiltration status of immune cells is also related to the clinical outcomes in ovarian carcinoma. The present research is aimed at constructing an immune-associated lncRNA signature with potential prognostic value for ovarian carcinoma patients.

**Methods:**

We obtained 379 ovarian carcinoma cases with available clinical data and transcriptome data from The Cancer Genome Atlas database to evaluate the infiltration status of immune cells, thereby generating high and low immune cell infiltration groups. According to the expression of the immune-associated lncRNA signature, the risk score of each case was calculated. The high- and low-risk groups were classified using the median risk score as threshold.

**Results:**

A total of 169 immune-associated lncRNAs that differentially expressed in ovarian carcinoma were included. According to the Lasso regression analysis and Cox univariate and multivariate analyses, 5 immune-associated lncRNAs, including AC134312.1, AL133467.1, CHRM3-AS2, LINC01722, and LINC02207, were identified as a predictive signature with significant prognostic value in ovarian carcinoma. The following Kaplan-Meier analysis, ROC analysis, and Cox univariate and multivariate analyses further suggested that the predicted signature may be an independent prognosticator for patients with ovarian carcinoma. The following gene set enrichment analysis showed that this 5 immune-associated lncRNAs signature was significantly related to the hedgehog pathway, basal cell carcinoma, Wnt signaling pathway, cytokine receptor interaction, antigen processing and presentation, and T cell receptor pathway.

**Conclusion:**

: This study suggested a predictive model with 5 immune-associated lncRNAs that has an independent prognostic value for ovarian carcinoma patients.

## 1. Introduction

Ovarian carcinoma is the 7th most common malignancies and the 8th leading cause of cancer-related death in women, with a 5-year survival rate less than 45% [1]. According to statistics in 2018, 295,414 new ovarian carcinoma cases and 184,799 ovarian carcinoma deaths occurred worldwide [2]. Due to its asymptomatic development, most cases are frequently diagnosed at an advance, incurable stage, leading to the highest mortality among gynecological malignancies [3, 4]. Biomarkers in ovarian carcinoma have attracted increasing attention so that they can monitor treatment response, detect recurrence, and detect disease at an earlier stage [5]. However, there are still lacks of reliable diagnostic markers and other diagnostic methods enabling detection at the early stage, as well as suitable for screening [6]. Considering that the presence of tumor-infiltrating lymphocytes in the ovarian carcinoma microenvironment has already been shown to be related to patients' survival [7], ovarian carcinoma is one of the first cancers that have been demonstrated to be associated with immune cell infiltration [8], screening reliable immune-related biomarkers is thought to be promising.

Over the past few years, a growing number of researchers have focused on a newly discovered type of noncoding RNA, termed long noncoding RNA (lncRNA), which refers to RNA transcripts of more than 200 bp that are incapable with protein coding [9]. It has been reported that lncRNAs involve in various important biological processes, including tumor progression, immune response, and tumorigenesis [10–12]. More importantly, dysregulated lncRNAs have been recognized to act as tumor oncogenes or suppressors in ovarian carcinoma and be associated with patients' survival. For example, HOTAIR is upregulated in ovarian carcinoma and is an independent prognosticator for the disease-free survival and overall survival in ovarian carcinoma patients [13].The downregulated lncRNAs BC004123 and BC007937 and the upregulated lncRNAs BC037530, AK021924, AK094536, AK094536, and BC062365 were found to be related to the survival and can be regarded as independent prognosticators for the patients with ovarian carcinoma [14]. The 5-year overall survival of patients with the high lncRNA AB073614 expression was inferior compared with patients with its low expression in ovarian carcinoma patients [15]. However, so far, the majority of studies have focused on lncRNAs as long RNA transcripts that do not encode proteins, making lncRNA to be a relatively poorly understood class of noncoding RNAs [16]. Emerging evidence has suggested that lncRNAs contribute to different phases of cancer immunity, including immune activation, infiltrating into cancer tissues, antigen presentation, antigen releasing, and immune cell migration [11]. Two lncRNAs AC104699.1.1 and RP11-284 N8.3.1 have been reported to exert protective effects throughout the progression of ovarian carcinoma and were related to the antitumor processes and activation of the immune system in the microenvironment [17]. A recent study showed that lncRNA SNHG12 facilitates immune escape of ovarian carcinoma cells by their crosstalk with M2 macrophages [18]. Therefore, it is reasonable to suggest that immune-associated lncRNAs may be the potential biomarkers for ovarian carcinoma patients and may serve as possible therapeutic targets. However, its role in the prognosis of ovarian carcinoma still needs to be further explored.

The development of bioinformatics provides convenience for solving these problems. Recently, gene expression datasets have been used to explore potential biomarkers, identify promising prognosticators, and determine valuable therapeutic targets for a variety of carcinomas. According to the gene expression profiles acquired from the public online, The Cancer Genome Atlas (TCGA) database, we performed the present study to identify immune-related lncRNAs in ovarian carcinoma using the bioinformatic tools and further establish an immune-associated lncRNA signature with prognostic value for patients with ovarian carcinoma, hoping to provide a promising predictor and some immune therapeutic targets.

## 2. Materials and Methods

### 2.1. Data Sources

The transcriptome data, lncRNA count data, and the clinical information of 379 ovarian carcinoma samples were obtained from the TCGA database. Moreover, a total of 88 adjacent normal cases were obtained from the Genotype Tissue Expression (GTEx) database for identifying differentially expressed lncRNAs.

### 2.2. Single-Sample Gene Set Enrichment Analysis (ssGSEA)

To explore the immune infiltration landscape of ovarian carcinoma samples, ssGSEA was utilized to quantify the infiltration levels of 29 immune cell types, based on the marker gene set for immune cell types that described by Bindea et al. [19]. The 379 ovarian carcinoma samples were classified into high and low immune cell infiltration groups (high group, *n* = 193; low group, *n* = 186) using hierarchical agglomerative clustering.

### 2.3. Verification of Immune Groups

The tumor purity, stromal score, immune score, and ESTIMATE score were evaluated using the “ESTIMATE” package [20]. Moreover, we assessed the relative gene expression levels of human leukocyte antigen (HLA) and CD274 (PD-L1) to validate the effectiveness of high and low immune cell infiltration groups. In addition, the Cell-type Identification by Estimating Relative Subsets of RNA Transcripts (CIBERSORT) algorithm served to determine the relative fraction of immune cells in the high and low immune cell infiltration groups [21].

### 2.4. Analysis of Differentially Expressed lncRNAs

In accordance with the expression profiles of lncRNA data from TCGA and adjacent normal ovarian samples from the GTEx database, differentially expressed lncRNAs were identified using the edge*R* package with the thresholds of |log_2_ fold chang (FC)  | >1 and false discovery rate (FDR) < 0.01. Using the same thresholds, differentially expressed lncRNAs in high and low immune cell infiltration groups were obtained according to the ovarian carcinoma cases. Venn diagram was used to select candidate lncRNAs.

### 2.5. Construction of an Immune-Associated lncRNA Prognostic Signature

Based on the clinical information of the ovarian carcinoma cases, the candidate lncRNAs were submitted for Cox univariate regression analysis with a criteria of *p* value less than 0.01. Subsequently, the Least Absolute Shrinkage and Selector Operation (LASSO) regression analysis was utilized to avoid overfitting the prognostic factors. Next, Cox multivariate regression analysis served to construct a prognostic signature. The prognostic risk score of each sample was calculated according to the expression level and the regression coefficient of the potential prognostic immune-related lncRNAs by the following formula [22]:

(1) $$\begin{array}{c} \text{Risk score}={\text{Expression}}_{\text{lncRNA}1}\times \beta \;{\text{coefficient}}_{\text{lncRNA}1}+{\text{Expression}}_{\text{lncRNA}2}\times \beta \;{\text{coefficient}}_{\text{lncRNA}2}+\cdots +{\text{Expression}}_{\text{lncRNAn}}\times \beta \;{\text{coefficient}}_{\text{lncRNAn}}. \end{array}$$

The *β* was calculated by log-transformed hazard ratio (HR) derived from Cox multivariate regression analysis [23]. LncRNAs with HR > 1 are risk factors for ovarian carcinoma, whereas lncRNAs with HR < 1 are protective factors. Ovarian carcinoma samples were grouped to high- and low-risk groups based on the median score as calculated. Survival curve was plotted by the Kaplan-Meier method. The time-dependent receiver operating characteristic (ROC) curve analysis was utilized to assess the accuracy of the model. Cox univariate and multivariate regression analyses were applied to assess the independent prognostic value of the risk score. In addition, the expression levels of key lncRNAs in ovarian carcinoma cell lines were evaluated using the European Bioinformatics Institute (EMBL-EBI, https://www.ebi.ac.uk) database, which is a user-friendly bioinformatic portal providing free and open access to a range of bioinformatic applications for sequence analysis [24].

### 2.6. Gene Set Enrichment Analysis (GSEA)

A GSEA served to identify the significant enrichment pathways correlated with the immune signature. GSEA was carried out using GSEA software (version 3.0, Cambridge, MA, USA). A *p* value less than 0.05 and FDR less than 0.25 indicated statistically significant.

### 2.7. Statistical Analyses

All statistical analyses were performed using the *R* version 3.5.1 (Institute for Statistics and Mathematics, Vienna, Austria). The *p* value less than 0.05 was considered to be statistically significant unless otherwise mentioned.

## 3. Results

### 3.1. Construction and Verification of High and Low Immune Cell Infiltration Groups in Ovarian Carcinoma

**Figure 1:**
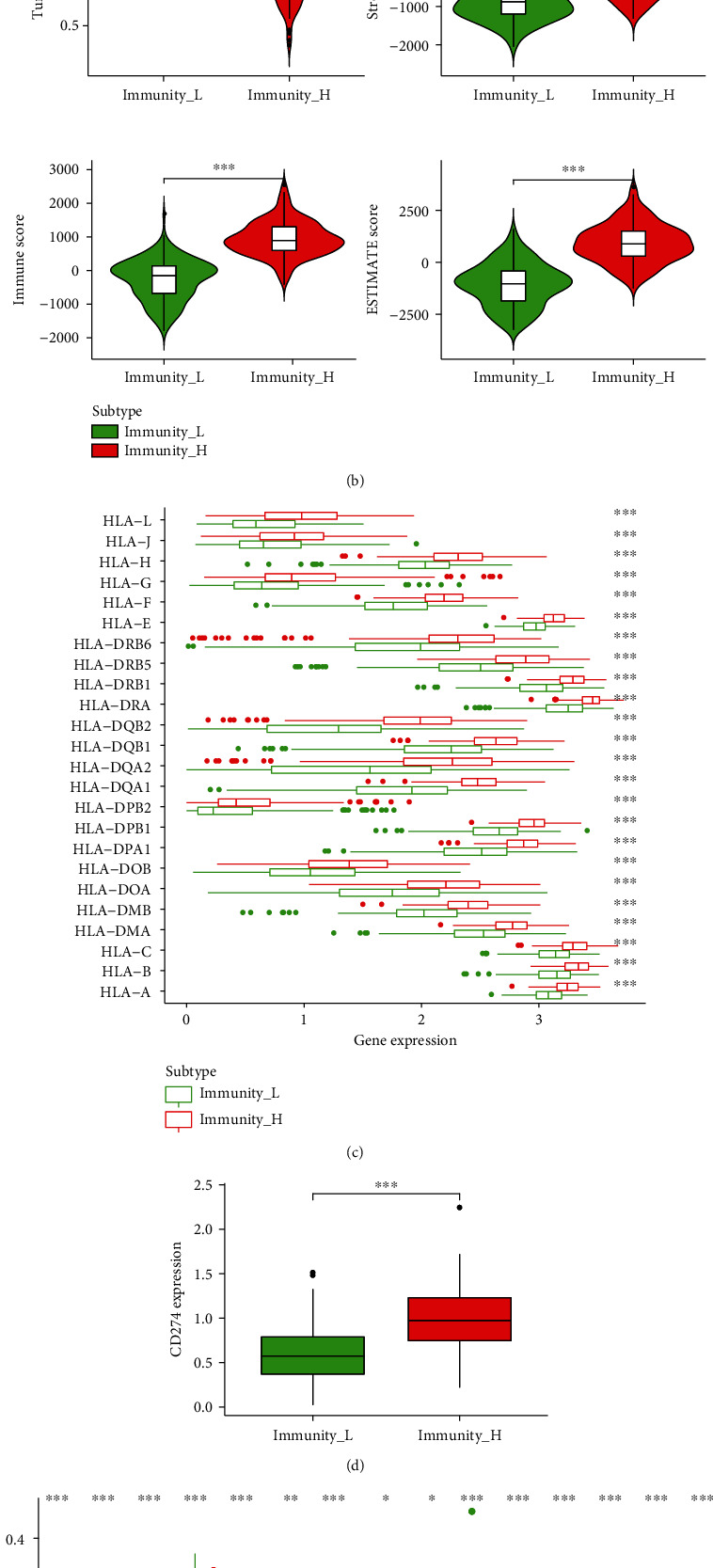
Construction of high and low immune infiltration groups in ovarian carcinoma. (a) Unsupervised clustering of ovarian carcinoma patients from the TCGA cohort using the ssGSEA method from immune cell types. The whole cohort was clustered into the high immune infiltration group (*N* = 193, immunity_H) and low immune infiltration group (*N* = 186, immunity_L). (b) The tumor purity, stromal score, immune score, and ESTIMATE score were assessed using the ESTIMATE algorithm between high and low immune infiltration groups. ^∗∗∗^*p* < 0.001. (c, d) The expression levels of HLA family members (c) and CD274 (d) were evaluated between high and low immune infiltration groups in the TCGA cohort. ^∗∗∗^*p* < 0.001. (e) The proportion difference of several immune cells in the two groups was assessed by the CIBERSORT method. TCGA: The Cancer Genome Atlas; HLA: human leukocyte antigen; ssGSEA: single-sample gene set enrichment analysis; N: number; CIBERSORT: Cell-type Identification by Estimating Relative Subsets of RNA Transcripts.

A total of 379 ovarian cases from TCGA were used to construct the immune infiltration groups. Twenty-nine immune-associated terms were included to eliminate the abundance of diverse immune cell types in ovarian carcinoma. The whole cohort was clustered into high immune infiltration group (*n* = 193) and low immune infiltration group (*n* = 186) using the unsupervised hierarchical clustering algorithm (Figure 1(a)), representing as the immune_H group and immune_L group, respectively. Next, the ESTIMATE algorithm was used to determine the feasibility of these two groups. Results shown in Figure 1(b) demonstrated that the immune_H group presented lower tumor purity and higher stromal score, immune score, and ESTIMATE score than those in the immune_L group, with significant differences (*p* < 0.001, Figure 1(b)). Moreover, we also observed that the expression levels of HLA family members and CD274 were significantly higher in the immune_H group when compared with the immune_L group (*p* < 0.001, Figures 1(c) and 1(d)). Also, the results indicated that the immune cell types in the immune_H group were more than in the immune_L group according to the CIBERSORT method (Figure 1(e)). Collectively, these results indicated that the immune grouping is desirable and can be used for the subsequent analysis.

### 3.2. Analysis of Differentially Expressed lncRNAs

**Figure 2:**
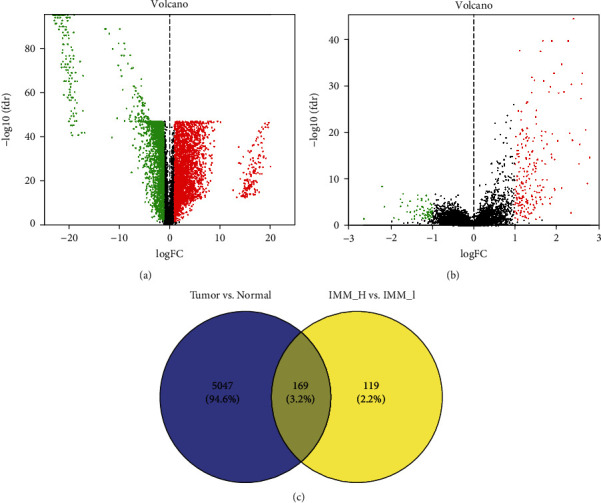
Analysis of differentially expressed lncRNAs. (a) The volcano plot of differentially expressed lncRNAs between ovarian carcinoma cases and adjacent normal cases. (b) The volcano plot of differentially expressed lncRNAs between high and low immune infiltration groups. (c) Venn diagram of (a) and (b). IMM_H represents high immune infiltration group; IMM_L represents low immune infiltration group.

In total, 5216 dysregulated lncRNAs, containing 3166 upregulated and 2050 downregulated lncRNAs, were obtained based on the data of 379 ovarian carcinoma cases and 88 adjacent normal cases with the thresholds of ∣ log_2_FC  | >1 and FDR < 0.01 (Figure 2(a)). Furthermore, we acquired 288 dysregulated lncRNAs in the immune_H group compared with the immune_L group, including 212 upregulated and 76 downregulated lncRNAs (Figure 2(b)). Finally, the Venn diagram identified 169 differentially expressed lncRNAs associated with immune infiltration (Figure 2(c)).

### 3.3. Establishment of the Immune-Associated lncRNA Signature

**Figure 3:**
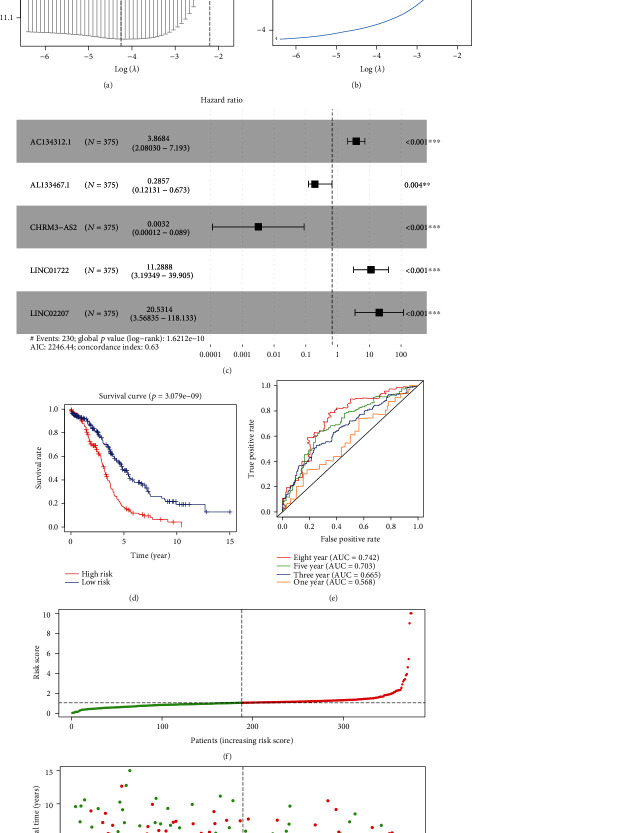
Construction of an immune-related lncRNA signature associated with the prognosis of ovarian carcinoma. (a, b) 12 immune-related lncRNAs were identified by a Lasso regression analysis. (c) Cox multivariate regression analysis of 5 immune-related lncRNAs for construction of a prognostic model in ovarian carcinoma. (d) Kaplan-Meier survival curve between high- and low-risk groups in the TCGA cohort. (e) Time-dependent ROC curve analysis for evaluating the reliability of the prognostic model. (f) The risk curve of each case reordered by risk score. (g) Scatter plot of the cases. Green dots presented survival cases, and red dots presented death cases. ROC: receiver operating characteristic.

Using the clinical information of the abovementioned 169 immune-associated differentially expressed lncRNAs, the Cox univariate regression analysis identified 12 dysregulated lncRNAs associated with the overall survival with the cutoff criteria of *p* value <0.01 (*p* < 0.01, Table 1). To avoid overfitting this analysis, these 12 immune-associated lncRNAs were submitted for a Lasso regression analysis, and the results showed that 11 dysregulated lncRNAs were related to ovarian carcinoma immune cell infiltration (Figures 3(a) and 3(b)). Subsequently, Cox multivariate regression analysis was carried out to construct the prognostic model. As shown in Figures 3(c), 5 immune-related lncRNAs were identified includingAC134312.1, AL133467.1, CHRM3-AS2, LINC01722, and LINC02207. Accordingly, risk score of each cases was calculated as follows: risk score = (1.353 × the expression level of AC134312.1) + (−1.253 × the expression level of AL133467.1) + (−5.741 × the expression level of CHRM3 − AS2) + (2.424 × the expression level of LINC01722) + (3.022 × the expression level of LINC02207).

The median risk score was utilized as a cutoff criteria to divide 379 ovarian carcinoma cases into high- and low-risk groups. The following Kaplan-Meier curve indicated that the overall survival of the high-risk group was significantly worse than that of the low-risk group (*p* < 0.001, Figure 3(d)). Moreover, the area under the curve (AUC) values of the time-dependent ROC curve were 0.568, 0.665, 0.703, and 0.742 for 1, 3, 5, and 8 years, respectively, demonstrating a good sensitivity of this prognostic signature (Figure 3(e)). With the prolongation of the survival time, the AUC values gradually increase, suggesting that this model may have a better prediction effect on the long-term survival for patients with ovarian carcinoma. Besides, as with the risk score increased, the mortality rate of ovarian carcinoma patients gradually raised (Figures 3(f) and 3(g)). In addition, the expression of these 5 immune-related lncRNAs in ovarian carcinoma cases and adjacent normal cases as well as their expression in immune_L and immune_H groups was presented in supplementary figure 1. The data showed that compared with adjacent normal cases, LINC01722 was upregulated, whereas AC134312.1, AL133467.1, CHRM3-AS2, and LINC02207 were downregulated in ovarian carcinoma (Supplementary figure 1a). LINC01722 was highly expressed in the immune_L group, whereas AC134312.1, AL133467.1, CHRM3-AS2, and LINC02207 were highly expressed in the immune_H group (Supplementary figure 1b). Also, we evaluated the expression of the 5 immune-related lncRNAs in ovarian carcinoma cells using the EMBL-EBI database. Except the absence of LINC01722 expression data, the expression of AL133467.1, CHRM3-AS2, and LINC01722 was downregulated in most detected ovarian carcinoma cell lines, which was consistent with our results from TCGA (Supplementary figure 2a-2c). Additionally, AC134312.1 was also downregulated in ovarian carcinoma OV7 cells (Supplementary figure 2d). Taken together, these results provided evidences for the 5 immune-related lncRNAs signature as a predictive model in ovarian carcinoma.

### 3.4. Prognostic Value of the 5 Immune-Associated lncRNAs Signature in Ovarian Carcinoma

**Figure 4:**
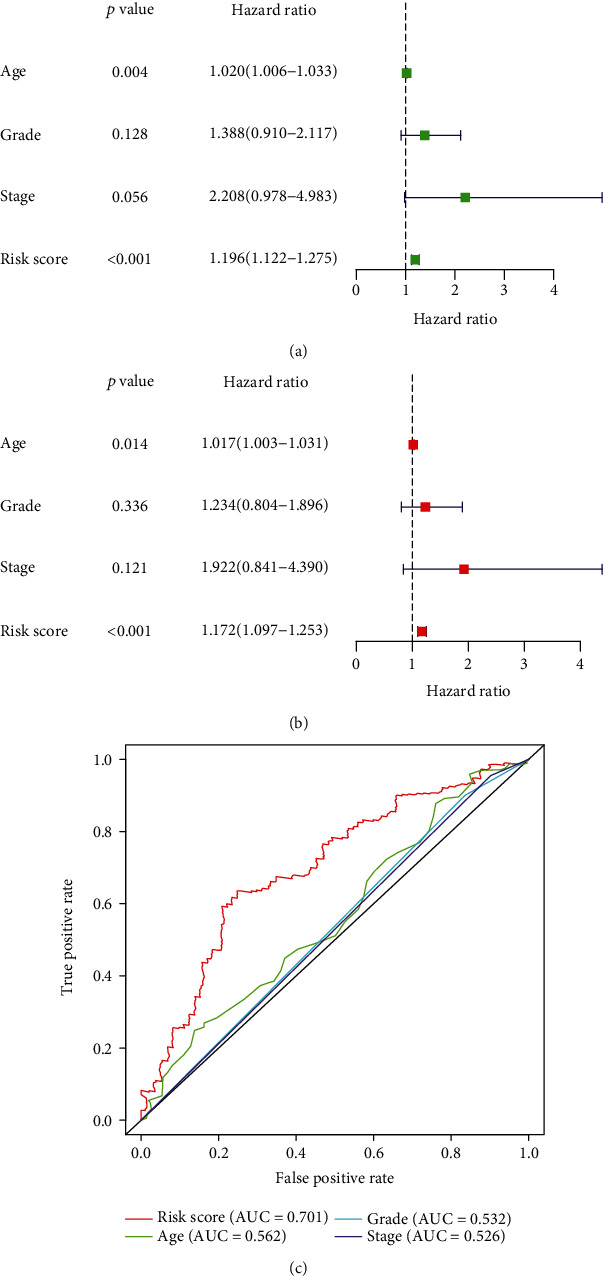
Independent prognostic values of the prognostic model. (a) Cox univariate analysis of the risk score and clinical features for overall survival in the TCGA cohort. (b) Cox multivariate analysis of the risk score and clinical features for overall survival in the TCGA cohort. (c) Calculation of the AUC for the risk score, age, grade, and stage using the ROC curve. TCGA: The Cancer Genome Atlas; AUC: area under the curve; ROC: receiver operating characteristic.

Cox univariate and multivariate regression analyses were used to determine the independent prognostic values of the risk score of the 5 immune-related lncRNA signature, age, histological grade, and pathological stage. The results shown in Figures 4(a) and 4(b) demonstrated that the risk score of this signature was the independently prognostic indicator for ovarian carcinoma patients. The time-dependent ROC curve showed that the AUC of the risk score of the 5 immune-related lncRNAs signature was 0.701, which was better than other clinical features (Figure 4(c)). All these results suggested that the risk score of the 5 immune-associated lncRNAs signature may be an independent prognosticator for patients with ovarian carcinoma.

### 3.5. Functional Analysis of the 5 Immune-Associated lncRNAs Signature

**Figure 5:**
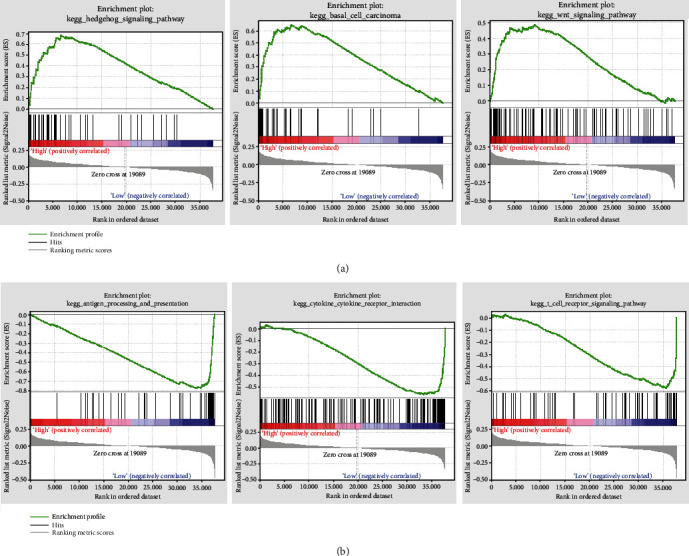
Results of GSEA in the TCGA cohort. (a) Three significantly enriched signaling pathways in the high-risk group. (b) Three significantly enriched signaling pathways in the low-risk group. GSEA: gene set enrichment analysis; TCGA: The Cancer Genome Atlas.

Furthermore, we also evaluated the function of the 5 immune-associated lncRNA signature using the risk score by GSEA. With the criteria *p* < 0.05 and FDR < 0.25, the high-risk score group was enriched into 7 significant signaling pathways, including the hedgehog pathway, basal cell carcinoma, and Wnt signaling pathway (Figure 5(a)), whereas the low-risk group was enriched into 20 signaling pathways, including cytokine receptor interaction, antigen processing and presentation, and T cell receptor pathway (Figure 5(b)).

## 4. Discussion

In present study, using 379 ovarian carcinoma cases with available clinical data and transcriptome data from the TCGA database, we assessed the infiltration status of immune cells to generate high and low immune cell infiltration groups. On the basis of the two groups, the differentially expressed lncRNAs associated with immune infiltration were identified. Then, we identified 5 immune-related lncRNAs, including AC134312.1, AL133467.1, CHRM3-AS2, LINC01722, and LINC02207 as a predictive signature with prognostic significance for ovarian carcinoma patients.

It is well documented that the immune system is a decisive factor in the development and progression of various human cancers [25]. Compelling evidences have suggested that the ovarian carcinoma is an immunogenic tumor and recognized and attacked by the immune system [26, 27]. The tumor microenvironment in ovarian carcinoma is comprised of an intricate system of immune cells, including macrophages, dendritic cell, T cell, and NK cell [28] The different types and numbers of infiltrating immune cells in different locations contribute to the high heterogeneity of the ovarian carcinoma immune microenvironment. In present research, using the transcriptomic data of 379 ovarian carcinoma cases, ssGSEA was applied to assess the infiltration status of immune cells to generate high and low immune cell infiltration groups. Then, we found that the high infiltration group has a higher stromal score, immune score, and ESTIMATE score and a lower tumor purity than the low immune infiltration group. We also performed the CIBERSORT algorithm to detect the expression of HLA family members and CD724 to verify the heterogeneity of the immune microenvironment in ovarian carcinoma. Finally, we obtained reasonable high and low immune infiltration groups in ovarian carcinoma.

With the development of bioinformatic analysis and gene expression profiles, including lncRNAs expression, from high-throughput sequencing, data are frequently used to investigate valuable immune-associated biomarkers to help identify potential prognostic targets [29, 30]. Moreover, lncRNA regulating the immune microenvironment of human ovarian carcinoma has gradually become one of the attractive parts in the field of RNA biology [11]. Numerous lncRNAs have been revealed to regulate the immune system in diverse ways, containing T-cell infiltration, immune cell activation/differentiation, and regulating antigen release [31]. Guo et al. reported that two lncRNAs, AC104699.1.1 and RP11-284 N8.3.1, were notably related to patients' survival and disease stage in ovarian carcinoma by activating the immune system response [17]. Two immune-related lncRNAs, FTX and LINC00665, were identified as prognostic biomarkers in high-grade serous ovarian carcinoma [32]. In present study, according to the Cox univariate analysis, Lasso regression analysis, and Cox multivariate analysis, we identified 5 immune-associated lncRNAs, including AC134312.1, AL133467.1, CHRM3-AS2, LINC01722, and LINC02207, as a potential prognostic signature in ovarian carcinoma using the data from TCGA. Among these lncRNAs, AC134312.1, LINC01722, and LINC02207 with *β* coefficient score > 0 were shown to be risk-associated lncRNAs, whereas AL133467.1 and CHRM3-AS2 with *β* coefficient score < 0 were identified as protective lncRNAs. We further observed that AC134312.1, AL133467.1, CHRM3-AS2, and LINC02207 were downregulated, whereas LINC01722 was upregulated in ovarian carcinoma compared with normal tissues. The low-expression genes included in the model may be due to their combined expression levels and prognostic values having a crucial effect on the prognosis of patients. Subsequently, to investigate the reliability of this predictive model, patients with ovarian carcinoma were classified into high- and low-risk groups according to the median risk score. The results revealed that the high-risk group presented a worse overall survival than that of the low-riskgroup, and the following time-dependent ROC analysis suggested that the 5 immune-associated lncRNAs signature have a better predictive effect on patients with longer survival time. Next, to evaluate the 5 immune-related lncRNAs in clinical application in ovarian carcinoma, we compared this established signature with the clinical features including age, grade, and stage using the Cox regression analyses and ROC analysis. Surprisingly, we verified that the 5 immune-associated lncRNAs signature may be an independent prognosticator for patients with ovarian carcinoma. The research on these 5 immune-associated lncRNAs has not been mined; so, we have no way to know the function of these lncRNAs. But in terms of the results of the GSEA, these lncRNAs may be closely related some pathways, such as Wnt signaling pathway and T cell receptor pathway, which still need to be further explored.

There are some disadvantages or limitations in this study because it is retrospective. The 5 immune-related lncRNA prediction model was constructed relying solely on the TCGA database. The experimental study was not performed to verify the expression of 5 immune-associated lncRNAs in ovarian carcinoma. Therefore, further experiments are needed to validate the results of this paper. In addition, due to the limited lncRNA chip of ovarian carcinoma in the GEO database, we did not identify the corresponding probes for the 5 immune-associated lncRNAs; thus, the prognostic significance of the signature has not been further verified and needs to be considered in the future.

In conclusion, we identified 5 immune-associated lncRNA signatures in ovarian carcinoma according to the cases downloaded from TCGA database. The 5 immune-associated lncRNA signatures were found to be an independent prognosticator for ovarian carcinoma patients. The signature provides a novel insight into immune-associated lncRNAs in ovarian carcinoma and identifies potential biomarkers for the prognosis and immunotherapy.

## Figures and Tables

**Table 1:** Cox univariate regression analysis of 12 immune-related lncRNAs according to the TCGA cohort.

ID	HR	HR.95L	HR.95H	*p* value
LINC01722	14.12659478	3.755973493	53.13154646	8.93*E*-05
CHRM3-AS2	0.002760515	0.000101978	0.074726302	0.000462978
AC134312.1	2.908726652	1.56875484	5.393252357	0.000700641
DLX2-DT	2.966227247	1.455983153	6.042998551	0.002747225
AL133467.1	0.289698151	0.127775502	0.656816194	0.003012605
USP30-AS1	0.781729557	0.66217333	0.922871809	0.003640291
LINC00702	2.128197121	1.26612018	3.577245713	0.004365151
LINC01281	0.012903175	0.000637549	0.261143691	0.00458325
LINC02207	10.93764457	1.847927508	64.73850746	0.008368776
LINC00996	0.299316761	0.121841982	0.735300932	0.008526547
AC134312.3	1.607494917	1.121588179	2.303911505	0.009743598
MYHAS	9.48698061	1.720690876	52.30620001	0.00979321

## Data Availability

The analyzed datasets generated during the study are available from the corresponding author on reasonable request. The RNA-Seq data have been deposited at the TCGA and GTEX databases.

## Supplementary Materials

Supplementary Materials Supplementary Figure 1: the expression of 5 immune-related lncRNAs in TCGA cohort. Supplementary Figure 2: the expression of AL133467.1, CHRM3-AS2, LINC02207, and AC134312.1 from the EMBL-EBI database.
