# Supplementary material for: Screening and Identification of an Immune-Associated lncRNA Prognostic Signature in Ovarian Carcinoma: Evidence from Bioinformatic Analysis
Source: Biomed Res Int. 2021 Apr 30;2021:6680036. doi: 10.1155/2021/6680036 (PMC8110384; doi:10.1155/2021/6680036)
Supplement: Supplementary Materials — Supplementary Figure 1: the expression of 5 immune-related lncRNAs in TCGA cohort. Supplementary Figure 2: the expression of AL133467.1, CHRM3-AS2, LINC02207, and AC134312.1 from the EMBL-EBI database. [file 6680036.f1.docx]

**Supplementary figures**

**Supplementary figure 1**

**
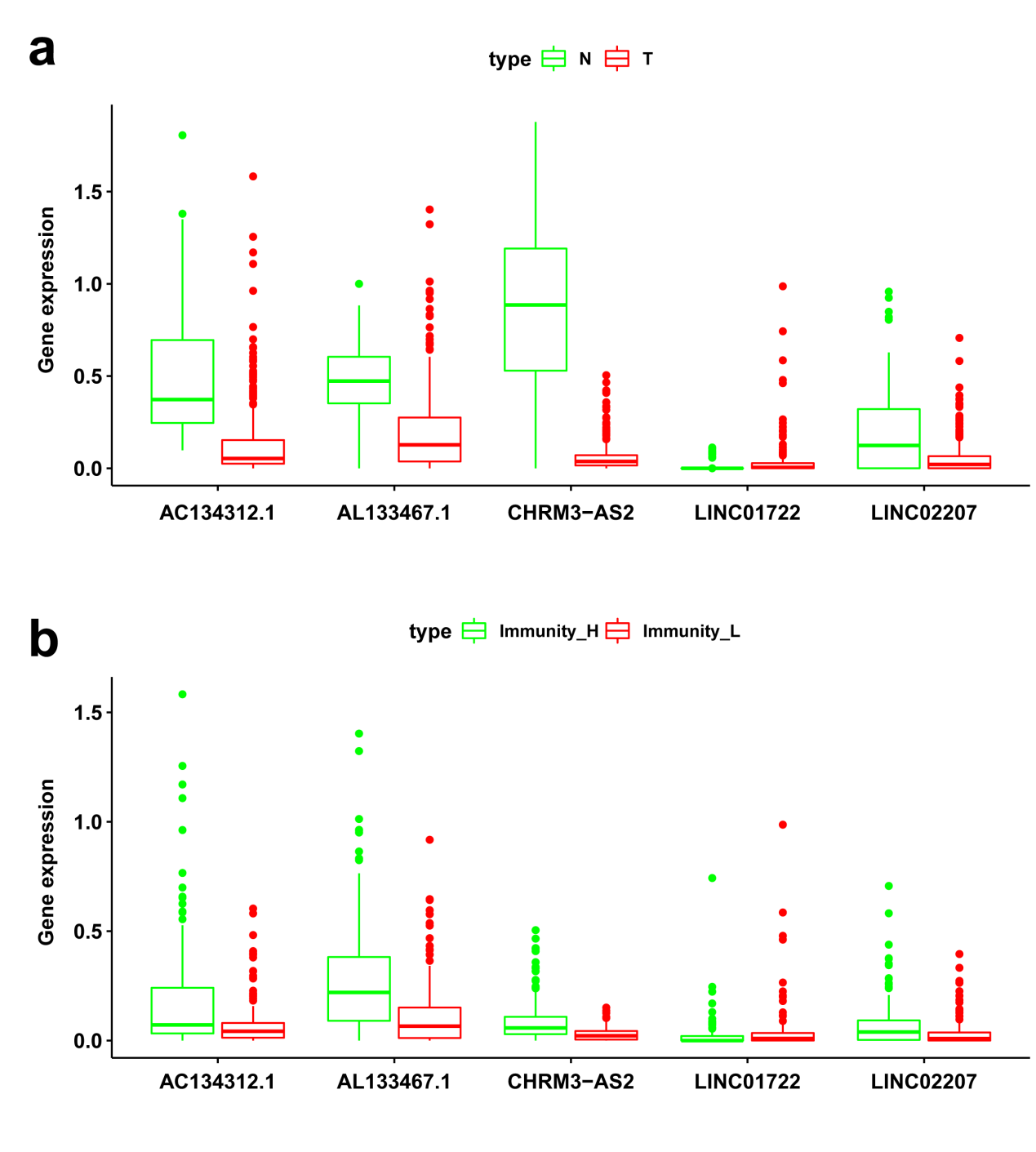
**

**Supplementary figure 1 The expression of 5 immune-related lncRNAs in TCGA cohort.**

(a) The expression of 5 immune-related lncRNAs between ovarian carcinoma cases and adjacent normal cases. (b) The expression of 5 immune-related lncRNAs between high and low immune infiltration groups.

**Supplementary figure 2**


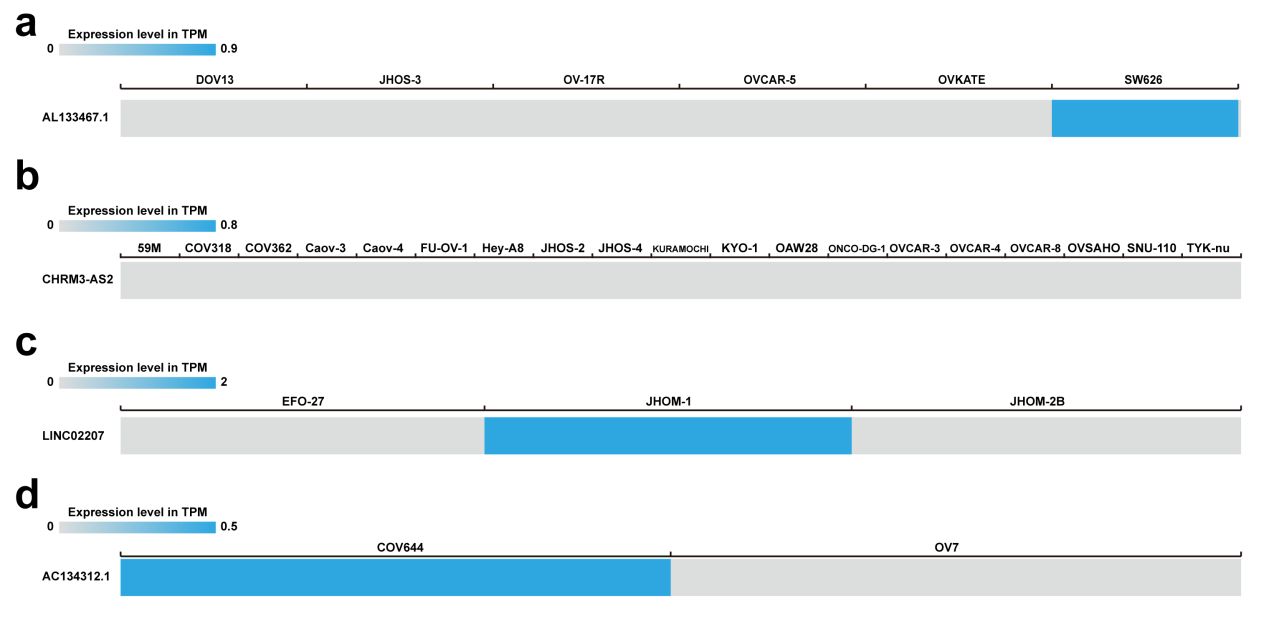


**Supplementary figure 2 The expression of AL133467.1, CHRM3-AS2, LINC02207, and AC134312.1 from EMBL-EBI database.** EMBL-EBI, European Bioinformatics Institute.
